# Umbilical cord mesenchymal stem cells combined with autologous platelet-rich plasma for lower extremity venous ulcers: A case report and literature review

**DOI:** 10.1097/MD.0000000000040433

**Published:** 2024-11-08

**Authors:** Linlin Jiao, Jing Nie, Limei Duan, Xiaoping Qiao, Yuanda Sui

**Affiliations:** a Nursing Department, Liaocheng People’s Hospital, Liaocheng, Shandong Province, China; b Department of Geriatrics, Liaocheng People’s Hospital, Liaocheng, Shandong Province, China; c Department of Critical Care Medicine, Liaocheng People’s Hospital, Liaocheng, Shandong Province, China; d Department of Traditional Chinese Medicine, Liaocheng People’s Hospital, Liaocheng, Shandong Province, China.

**Keywords:** human umbilical cord mesenchymal stem cells, lower extremity venous ulcer, platelet, rich plasma, treatment

## Abstract

**Rationale:**

Nonhealing ulcers are difficult to manage because they deviate from the normal wound healing process. Conventional therapy cannot achieve satisfactory therapeutic effects. To verify the effectiveness of combined treatment with human umbilical cord mesenchymal stem cells (hUMSCs) and platelet-rich plasma (PRP) for nonhealing ulcers, we studied a patient with left lower limb venous ulcer (LEVU) treated with combined injection therapy.

**Patient concerns:**

We present the case of a LEVU patient who has not healed for a long period of time (up to 1 year).

**Diagnoses:**

LEVU was diagnosed with clinical symptoms.

**Interventions:**

The hUMSCs plus PRP were injected into the wound edge and base (1 µL of cells/cm^2^ of wound surface), 0.5 mL at each point, with a distance of approximately 1 to 3 cm between points. The injection point was determined according to the extent of wound involvement.

**Outcomes:**

Seven days after hUMSC + PRP application, the wound area decreased by nearly 50%. The ulcers had almost completely healed by day 62, and no serious treatment-related toxic side effects were observed.

**Lessons:**

hUMSCs can improve wound healing through re-epithelialization, increased angiogenesis, and granulation tissue formation. PRP has also been suggested to promote wound healing through the secretion of various nutritional factors. The combination of hUMSCs and PRP has a mutually reinforcing effect, which may achieve a 1 + 1 > 2 effect. Therefore, the combination of hUMSCs and PRP may be a safe and effective treatment option for LEVU.

## 1. Introduction

Lower extremity venous ulcer (LEVU) is a serious and refractory manifestation of chronic venous insufficiency of the lower extremities. The incidence rate among the general population is as high as 1.1% to 1.8%.^[1,2]^The pathological and physiological basis of venous ulcers is lower limb venous hypertension, which can be caused by either obstructed venous reflux or venous reflux.^[3,4]^ Patients often experience swelling, dampness, and decay in their calves and ankles, with foul-smelling pus and dirty gray decaying material covering the wounds. Over the years, wounds have been difficult to close, and even if closed, patients are prone to recurrence, seriously affecting patients’ physical and mental health and daily life.^[5,6]^ Most patients require long-term treatment, wound cleaning, and dressing changes, which impose a serious economic and psychological burden and result in poor treatment outcomes.^[7,8]^ Therefore, promoting ulcer recovery and shortening treatment time are now becoming the focus of clinical research. At present, nonsurgical therapies, including traditional Chinese medicine, physical therapy, and cytokine therapy, are commonly used for the treatment of lower limb venous ulcers. However, the results of clinical studies on lower limb venous ulcers in the literature are not consistent.^[9,10]^

Human umbilical cord mesenchymal stem cells (hUMSCs) represent an attractive stem cell source for tissue regeneration in clinical practice.^[11]^ hUMSCs play a positive role in regulating the inflammatory environment, promoting angiogenesis, facilitating the migration of keratinocytes, and recruiting other host cells, which contributes to the production of granulation tissue, promotes angiogenesis and re-epithelialization, improves wound healing and attenuates scar formation.^[12,13]^ MSCs can secrete large amounts of soluble factors to promote the growth, survival, and function of wound repair cells.^[11]^ Autologous platelet-rich plasma (PRP) is an emerging clinical treatment technique that has been widely applied in many research fields and has been proven to promote wound repair.^[2,14]^ PRP contains high concentrations of platelets, white blood cells, and fibrin, which regulate the administration of growth factors and cell proliferation and migration in wound tissue, potentially improving chronic wound healing.^[2,14,15]^ PRP contributes to accelerating the healing of chronic wounds and promotes regeneration and repair processes at the wound surface.^[15]^ Basic studies also indicate that PRP can promote the migration, proliferation, angiogenesis, and tissue synthesis metabolism of MSCs.^[16–19]^

According to ClinicalTrials.gov, to date, 25 clinical trials of hUMSCs or PRP for pressure injury have been registered worldwide and have preliminarily proven the safety and feasibility of the use of hUMSCs or PRP for treating pressure injury. However, cases of pressure injury treated with PRP combined with hUMSCs have seldom been reported. Here, we report a patient with a lower extremity venous ulcer who has not healed for a long period of time (up to 1 year). We hope that the results of this study can provide a preliminary theoretical basis for the combination of hUMSCs and PRP in the treatment of LEVU.

## 2. Case report

### 2.1. Case presentation

The patient is a 70-year-old male who has been suffering from left lower extremity venous ulcers for more than 1 year. The size of the lesion was 15 × 4 × 1 cm, with 75% yellow tissue, 25% red granulation tissue, a moderate amount of light yellow exudate, and an odor. The surrounding skin showed impregnation, pigmentation, and scarring. The laboratory findings were as follows: serum albumin, 32 g/L; hemoglobin, 112 g/L; red blood cell count, 4.3 × 10^12^/L; and white blood cell count, 11.2 × 10^9^/L. The specific treatment method is unknown. Owing to the inability of the wound to heal, the patient went to Liaocheng People’s Hospital for treatment on October 14, 2022. Considering the poor efficacy of conventional therapies, hUMSC plus PRP therapy was proposed under the advice and guidance of the specialist group. The patient and their family agreed to the method and signed an informed consent form. The therapeutic scheme was then discussed and approved by the ethics committee of the hospital.

### 2.2. Platelet-rich plasma preparation

Whole venous blood (40 mL) was collected from the forearm vein, which was used for PRP production using the improved 2-step centrifugation method described earlier.^[20]^ The preparation process of PRP includes 2 centrifugation steps (300 × g for 5 minutesand 700 × g for 17 minutes). After 2 steps, the platelet-poor plasma was removed to obtain approximately 5 mL of PRP, which was used to resuspend hUMSCs for subsequent use.

### 2.3. Treatment with hUMSCs plus PRP

hUMSCs (P4) were suspended in 5 mL of PRP, and the total number of transplanted cells was 5 × 10^6^. hUMSC injection was performed in 4 steps. Before cell therapy, physiological saline was used for thorough debridement of ulcers, and if necessary, it was combined with sharp debridement. Clinicians performed the hUMSC injection, in which hUMSCs plus PRP were injected into the wound edge and base (1 µL cells/cm^2^ of the wound surface), 0.5 mL at each point, with a distance of approximately 1 to 3 cm between points. The injection point was determined according to the extent of wound involvement. After a single dose of hUMSCs and PRP was applied, the wound was covered with a nonadherent and transparent dressing. During hUMSC plus PRP therapy, local creams or dressings with potential healing effects are not allowed. The patient was assessed by the investigators after receiving the investigational product.

## 3. Results

### 3.1. Safety assessment

The patient’s vital signs remained stable after treatment. No acute treatment-related allergic reactions or serious adverse reactions were observed except for slight bleeding, exudation, and mild swelling at the injection site. Similarly, no delayed hypersensitivity or secondary infections were detected after treatment. The mild swelling disappeared after 2 days without any intervention.

### 3.2. Efficacy evaluation

**Figure 1.**
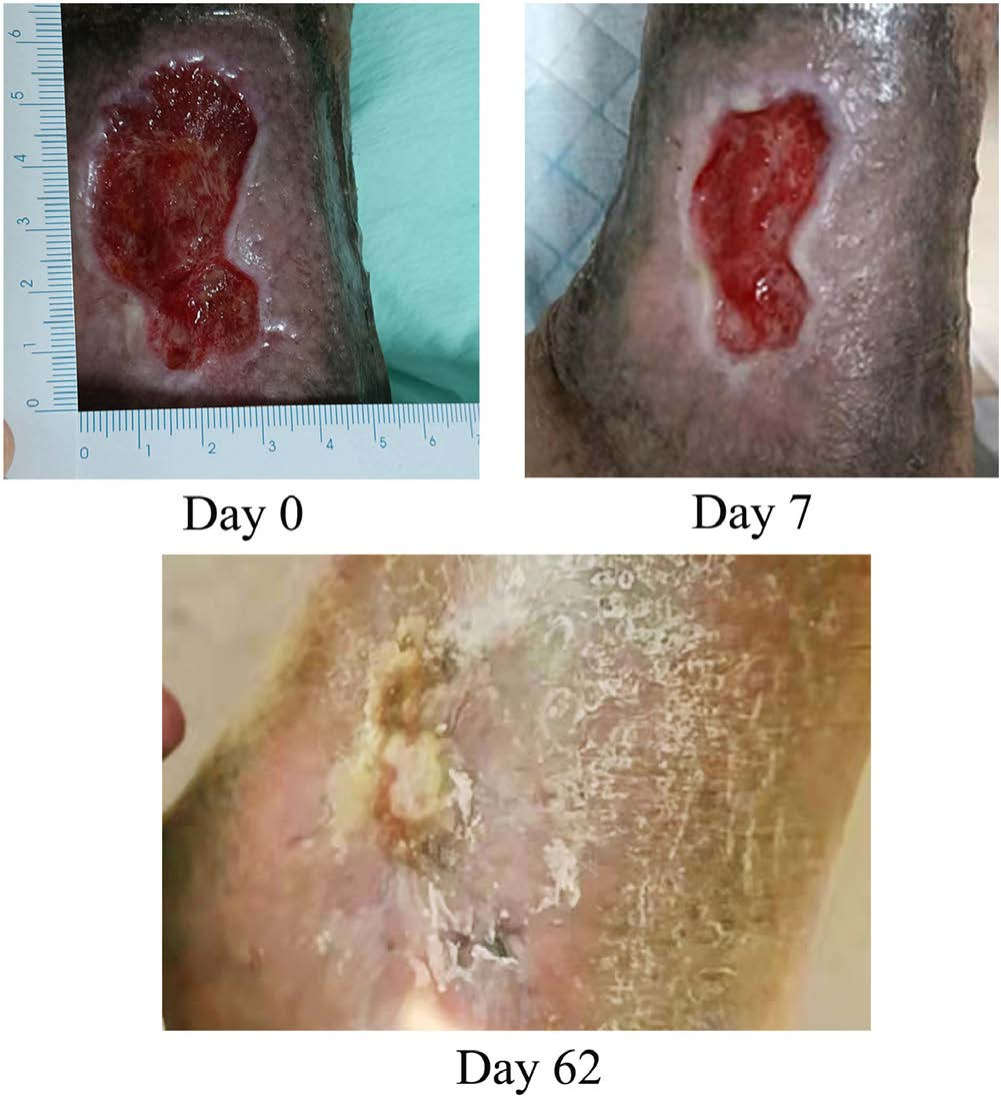
Representative photos showing the healing progress of lower extremity venous ulcer on days 0, 7 and 62 after cell treatment.

On postinjection Day 4, the wound surface had not changed. The size was 5 × 4 × 1 cm and the wound base was red in color. The granulation tissue was normal. A mild yellow exudate was found, without odor. The edge of the wound was clear. On postoperative Day 7, the wound area decreased by 50%, from 28.182 to 14.25 cm^2^. The digital images of Days 0, 7 and 62 are shown in Figure 1. The push score decreased from 16 to 12 points. After discharge, the patient was followed up via Chinese remote nursing combined with the family visit mode. The wound healing time was 62 days.

## 4. Discussion

Pressure injury, a health problem with continuously increasing prevalence and incidence, seriously affects patients’ quality of life. If conventional treatment does not yield effective results, it is highly likely to evolve into a chronic disease. A reduced local growth factor content, accelerated degradation or reduced trauma activity, and the presence of apoptosis are important factors in the difficulty of healing stress injuries. Therefore, there is an inevitable trend toward the adoption of growth factor therapy for the treatment of pressure injuries.

**Figure 2.**
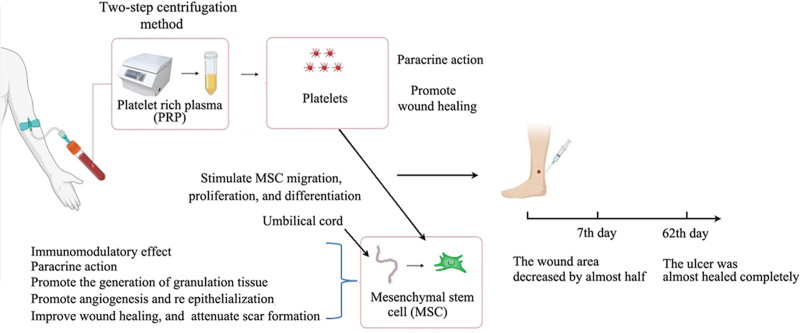
The mechanism of combined action of hUMSCs and PRP treatment for ulcer healing. hUMSCs = human umbilical cord mesenchymal stem cells, PRP = platelet-rich plasma.

PRP may act as a regulatory factor for cell migration and wound healing, promoting the migration of MSCs and the process of wound repair. The mechanism of the combined action of hUMSCs and PRP for lower extremity venous ulcer healing is shown in Figure 2. PRP may provide a suitable microenvironment for MSCs to promote their proliferation and differentiation.^[15–18]^ The combination of MSCs and PRP may also increase the number of fibroblasts and keratinocytes in the wounded skin.^[21,22]^ Fibroblast healing, remodeling of injured skin, and migration of keratinocytes can promote re-epithelialization through the healing process.^[23]^ Lian et al.^[24]^ evaluated the combined effect of MSCs and PRP and reported that the wound healing rate of the MSC plus PRP group was significantly greater than that of the other groups. The expression of TGF-β1 in the MSC plus PRP group was 1.5 times and 1.4 times greater than that in the PRP and MSC alone groups, respectively.^[22]^ This increased expression may promote the synthesis of collagen and integrins in fibroblasts and increase the migration of epithelial cells.^[22]^ MSCs and PRP can induce stronger angiogenesis responses in wound healing. PRP combined with MSCs may represent a promising approach to the treatment of wound healing.^[23,24]^

In this study, we report a case of a patient with a lower extremity venous ulcer who has not healed for a long period of time (up to 1 year). The safety and efficacy of intradermal injection of hUMSCs and PRP in accelerating wound healing were evaluated. No significant side effects were found with cell injection therapy, indicating good tolerance and safety. Our research results indicated that the administration of hUMSCs can improve granulation, promote re-epithelialization, and accelerate wound closure. Compared with traditional treatment, MSC treatment can shorten the healing time of ulcers. The healing time of LEVU varies greatly depending on the stage of LEVU and the patient’s physical condition.

There are several limitations to this study. First, this study reported only 1 patient, and a placebo group was not designed for ethical reasons. Second, owing to the patient’s difficulty in movement, the imaging data of wound recovery were taken with the assistance of the patient’s family, and the ruler tools were not strictly applied. Finally, the benefits of combination therapy cannot be well demonstrated due to the absence of a separate PRP and hUMSC treatment group. Given these limitations, we will further validate these issues in subsequent trials.

## 5. Conclusions

Combined treatment with hUMSCs and PRP can improve and accelerate the LEVU healing process. hUMSCs and PRPs have great therapeutic potential in wound healing. Further in-depth analysis and exploration of the mechanism of action between hUMSCs and PRP will contribute to optimizing therapeutic strategies for patients with nonhealing ulcers.

## Author contributions

**Resources:** Linlin Jiao, Jing Nie, Xiaoping Qiao.

**Supervision:** Yuanda Sui.

**Writing – original draft:** Linlin Jiao, Jing Nie.

**Writing – review & editing:** Limei Duan, Yuanda Sui.
